# An intersectional analysis of LncRNAs and mRNAs reveals the potential therapeutic targets of Bi Zhong Xiao Decoction in collagen-induced arthritis rats

**DOI:** 10.1186/s13020-022-00670-z

**Published:** 2022-09-16

**Authors:** Cailin He, Yang Wang, Yuqi Wen, Teng Li, En Hu, Siqing Zeng, Bo Yang, Xingui Xiong

**Affiliations:** 1 Institute of Integrative Medicine, Department of Integrated Traditional Chinese and Western Medicine, Xiangya Hospital, Central South University, Changsha, 410008 China; 2 National Clinical Research Center for Geriatric Disorders, Xiangya Hospital, Central South University, Changsha, Hunan P.R. China 410008

**Keywords:** Long non-coding RNA (lncRNA), Messenger RNA (mRNA), Bi Zhong Xiao decoction, Collagen-induced arthritis, Transcriptomics, Rheumatoid arthritis, Traditional Chinese medicine

## Abstract

**Background:**

Bi Zhong Xiao decoction (BZXD), a traditional Chinese herbal formula, has been used clinically for many years to treat rheumatoid arthritis (RA). Both clinical and experimental studies have revealed that BZXD is effective in treating RA, but the mechanism remains unclear. In this study, we aimed to explore the mechanism of efficacy of BZXD through transcriptomic analysis of lncRNA and mRNA.

**Methods:**

The combination method of ultra-high performance liquid chromatography-mass spectrometry/mass spectrometry was used to assess the quality of BZXD. The efficacy of BZXD in treating collagen-induced arthritis (CIA) was evaluated by clinical assessment, weight changes, hematoxylin–eosin and safranin o-fast green staining, and Micro-CT. Arraystar rat lncRNA-mRNA chip technology was used to determine the lncRNA and mRNA expression profiles of the Control, CIA and BZXD groups, and to screen gene expression profiles related to the curative effect of BZXD. A lncRNA-mRNA co-expression network was constructed for the therapeutic efficacy genes. Through GO function and KEGG pathway enrichment analysis, the biological functions and signaling pathways of therapeutic efficacy genes were determined. Based on fold change and functional annotation, key differentially expressed lncRNAs and mRNAs were selected for reverse transcription-quantitative polymerase chain reaction (RT-qPCR) validation. The functions of lncRNAs targeting mRNAs were verified in vitro.

**Results:**

We demonstrated that BZXD could effectively reverse bone erosion. After BZXD treatment, up to 33 lncRNAs and 107 mRNAs differentially expressed genes were reversely regulated by BZXD. These differentially expressed lncRNAs are mainly involved in the biological process of the immune response and are closely related to the ECM-receptor interaction, MAPK signaling pathway, Focal adhesion, Ras signaling pathway, Antigen processing and presentation, and Chemokine signaling pathway. We identified four lncRNAs (uc.361−, ENSRNOT00000092834, ENSRNOT00000089244, ENSRNOT00000084631) and three mRNAs (Acvr2a, Cbx2, Morc4) as potential therapeutic targets for BZXD and their microarray data consistent with the RT-qPCR. In vitro experiments confirmed that silencing the lncRNAs ENSRNOT00000092834 and ENSRNOT00000084631 reversed the expression of target mRNAs.

**Conclusions:**

This study elucidates the possible mechanism of BZXD reversing bone erosion in CIA rats from the perspective of lncRNA and mRNA. To provide a basis and direction for further exploration of the mechanism of BZXD in treating RA.

**Supplementary Information:**

The online version contains supplementary material available at 10.1186/s13020-022-00670-z.

## Introduction

Rheumatoid arthritis (RA) is one of the most prevalent progressive autoimmune diseases [1]. The pathological feature of RA is a chronic and continuous development process, proliferative synovitis, synovial pannus formation, and the progressive destruction of cartilage and bone, which ultimately leads to progressive disability and a catastrophic decline in quality of life [2]. The main treatment goal of RA patients is still clinical remission, and low disease activity is the best option possible [3]. This means that patients need to fight the disease for a long time, and choosing safe, effective, and cheap medicine has become an important consideration for the treatment of RA. Traditional Chinese medicine (TCM) has been employed to treat RA for thousands of years [4–6]. These ancient therapies are still in use and development and have the advantages of low toxicity, relatively low price, and few side effects [7–9].

Bi Zhong Xiao decoction (BZXD), a representative TCM formula for RA treatment with promising clinical efficacy, has no significant side effects [10, 11]. Our previous studies have shown that BZXD has the effects of clearing away heat and detoxification, promoting blood circulation and dredging collaterals, and down-regulating the levels of pro-inflammatory factors TNF-α and IL-1β [12, 13]. We also investigated paeoniflorin (PA) as a bioactive component of the radix Paeonia alba, ferulic acid (FA), and p-Coumaric acid (p-CA) as the active compounds in Oldenlandia Diffusa (OD), these components absorbed into rat plasma and reduced the levels of inflammatory cytokines IL-1β and TNF-α, thereby inhibiting inflammation in CIA rats [14–16]. Proteomics analysis reveals that PA exerts its anti-inflammatory effect by decreasing two key proteins, leukemia inhibitory factor receptor (LIFR) and asporin (ASPN) [17]. However, TCM formulas are usually composed of several medicines containing hundreds of components. These components are mixed and interacted to affect an unknown number of cellular targets, concurrently generating systematic effects [18]. The synergistic and holistic philosophy of BZXD formulae in treating RA remains unknown.

More than 90% of the eukaryotic genome is involved in transcription [19], but only 2% of transcripts encode proteins [20], and the remaining 98% of transcripts that do not encode proteins are called non-coding RNAs (ncRNA) [21]. LncRNAs are non-coding RNA with lengths greater than 200 nucleotides and have almost no protein-coding ability [22]. LncRNAs actively participate in many important biological processes by controlling gene expression at the epigenetic, transcriptional, and post-transcriptional levels, and serve as the main regulatory factor that affects the expression levels of dozens or even hundreds of target genes [23–25]. Accumulating evidence suggests that lncRNAs play key roles in physiological and pathophysiological processes and are involved in the pathogenesis of RA [26, 27]. Furthermore, studies have shown that targeting these lncRNAs can treat most autoimmune diseases [28, 29]. Triptolide played a therapeutic role in the CIA rats by inactivating lncRNA RP11-83J16.1 mediated URI1 and β-catenin signaling [30]. Shikonin has anti-inflammatory properties and the ability to mediate cellular and humoral immunity, which can promote the acetylation of histone H3 at the promoter of LncRNA NR024118, thereby inhibiting the inflammatory response in CIA mice 26640499 [31]. In addition, a novel immunosuppressant (5R)-5-hydroxytriptolide (LLDT-8) also showed good efficacy on RA-FLS through the lncRNA WAKMAR2/miR-4478/E2F1/p53 axis [29].

Although more and more lncRNAs have been identified and included in the database, the functional characterization of lncRNAs is still in its infancy. Only a few lncRNAs have received good functional annotation [32, 33]. The uncharacterized lncRNA can be annotated through the systematic analysis of lncRNA-mRNA correlation [34]. For instance, Qin et al. [35] comprehensively analyzed the lncRNA and mRNA expression profiles in plasma of RA patients. Wu et al. [36] identified a novel biomarker lncRNA E2F3-IT1 for RA by integrating lncRNA-mRNA co-expression network analysis. Therefore, it is rational to believe that the analysis of co-expressed lncRNAs and mRNAs helps to capture a more comprehensive regulatory network and gain a clearer understanding of the regulatory mechanisms of BZXD formulae [23, 37, 38].

In this study, we use the transcriptomics analysis of lncRNAs and mRNAs to elucidate the multifaceted mechanism of the BZXD formulas by determining the interactions and exact therapeutic targets. These findings will provide a more comprehensive perspective and insight into the mechanism of BZXD against RA.

## Material and methods

### BZXD preparation

The original composition of BZXD is listed in Table 1, herbs were combined in a ratio of 30:30:30:30:15:15:15:10:5 at the dry weight. Dried crude herbs were obtained from the Pharmacy of Xiangya Hospital, Central South University, and the quality was in accordance with the People's Republic of China Pharmacopoeia (2020). All raw herbal voucher specimens are deposited in the author's laboratory and identified by Professor Hu Suiyu, Department of Chinese Herbal Medicine, Central South University (Changsha, China). We calculated the daily dose of BZXD herbal medicine in humans and converted it to the required daily dose in rats using the body surface area of an individual weighing 70 kg as previously described [39]. These crude drugs were immersed in distilled water for 1 h and boiled twice at 100 °C (the amount of water added was 10 times and 8 times of crude drug). Finally, the two filtrates were combined to make a concentrated decoction, which was sealed and stored in a refrigerator at 4 °C for later use.

**Table 1:** Components of Bi Zhong Xiao decoction

Latin name	Chinese name	Plant name	Medicinal part	Batch number	Ratio
*Hedyotis Diffusae Herba*	Baihuasheshecao	*Hedyotis diffusa Willd*	Whole Herb	21030607	30
*Herba Sarcandrae*	Zhongjiefeng	*Sarcandra glabra (Thunb.) Nakai*	Whole Herb	21042008	30
*Actinidia Chinensis Planch*	Mihoutaogen	*Actinidia arguta (Sieb. et Zucc) Planch. ex Miq*	Root	19010314	30
*Coicis Semen*	Yiyiren	*Coix lacryma-jobi L.var.mayuen (Roman.) Stapf*	Seed	21051910	30
*Radix Salviae Miltiorrhizae*	Danshen	*Salvia miltiorrhiza Bunge*	Root	21062309	15
*Trachelospermumjasminoides*	Luoshiteng	*Trachelospermum jasminoides (Lindl.) Lem*	Leaf and Stem	21011901	15
*Paeoniae Radix Alba*	Baishao	*Paeonia lactiflora Pall*	Root	20210802	15
*Impatiens Balsamina*	Fengxiantougucao	*Speranskia tuberculate (Bunge) Baill*	Whole Herb	21051803	10
*Radix Glycyrrhizae*	Gancao	*Glycyrrhiza uralensis Fisch*	Root	21041905	5

### UPLC–MS/MS analysis

An Ultra-Performance Liquid Chromatography-Tandem Mass Spectrometry (UPLC–MS/MS) analysis was conducted to identify the main chemical components of the BZXD preparation. The BZXD concentration was 2 g/ml (a 1 ml solution containing 2 g of the original herbs). The standards for Paeoniflorin (Lot: M28GB143089), Rosmarinic acid (Lot: Y16A9K67403), Salvianolic acid B (Lot: P13N11F130912), Glycyrrhizic acid (Lot: Y02J11L113432), Ferulic acid (Lot: G13S11L124423), and p-Coumaric acid (Lot: Y30A11C112277) were provided by Yuanye Bio-Technology Co., Ltd (purity  ≥ 98%, Shanghai, China).

The composition of phytochemicals in BZXD was determined using AB Sciex API 4000 LC/MS/MS system, the chromatographic column was Agilent AdvanceBio Glycan Map (2.1 × 150 mm, 2.7 μm). In addition, the mobile phase in this study, consisting of 2 mmol/l ammonium acetate water (A) and acetonitrile (B), was employed to separate the analyte using a linear gradient program with a flow rate of 0.30 ml/min. Then, the post time was 20 min. The injection volume of each needle was 10 µl, and the column temperature was maintained at 40 °C temperature. The general MS parameters were optimized as follows: curtain gas 35 arb, collision gas 7 arb, IonSpray voltage negative −4500 V, temperature 400 °C, IonSource gas1 40 arb, IonSource gas 250 arb. Detection and quantification were carried out using negative-ion electrospray tandem mass spectrometry via multiple reaction monitoring (MRM).

In short, the processed samples were injected and tested. Confirm the retention position of the target peak according to the standard addition experiment, and obtain the peak area data. Finally, the content of the compound was calculated according to the ratio of the peak area of the standard to the peak area corresponding to the retention time and dilution factor in the sample.

### Experimental of CIA rats

Male specific-pathogen-free Sprague Dawley rats (6–7 weeks, 180–220 g) were supplied by the Laboratory Animal Centre of Central South University (Changsha, China). All rats received food and water at liberty and were housed in a 12 h dark/light cycle at 25 °C for at least 7 days to adapt to the environment. All experimental protocols were implemented following the Guide for the Care and Use of Laboratory Animals of the National Institutes of Health (NIH Publication No. 85–23, revised 1996) and were approved by the Animal Ethics Committee of Central South University (No. 2020sydw0898).

The rats were randomly divided into three groups (n = 8 each group): Control, CIA, and CIA + BZXD-treated (BZXD) group. CIA model rats were induced with the Protocol For the Successful Induction of Collagen-Induced Arthritis, bovine type II collagen (BIIC, 2 mg/ml, Chondrex, Inc, Washington DC, USA) was completely mixed with complete Freund's adjuvant (CFA,1 mg/ml, Sigma-Aldrich Co., St Louis, MO, USA). Rats were given intradermally with 200 μg collagen-CFA emulsion for primary immunization at the base of the tail. After 7 days, rats were injected 100 μg of collagen-incomplete Freund's adjuvant (IFA, 1 mg/ml, Sigma-Aldrich Co., St Louis, MO, USA) emulsion for enhanced immunization. After 14 days, the BZXD group rats were administrated BZXD once a day at a dose of 16.2 g/kg for 28 days; the Control and CIA groups rats were treated with an equal volume of saline solution.

### Arthritis assessments

Clinical assessment and the bodyweight changes were evaluated after arthritis onset. At least one entire foot was swollen, to be considered a successful arthritis induction. We recorded the clinical scoring to evaluate the severity of arthritis (maximum 12 scores per rat). More details of clinical scores were provided in our previous work. The rats were weighed on days 0, 7, 14, 21, 28, 35 and 42, and the percentage of weight change was calculated.

### Sample collection

On day 42, all rats were anesthetized by intraperitoneal injection of 3% pentobarbital sodium (60 mg/kg) and sacrificed by neck dislocation after deep anesthesia. After sequentially removing the skin, muscle, adipose tissue, bone, and tendon at the knee joint of the rat, we separated the synovial tissue from the hind paw with a scalpel and stored it at −80 °C. To evaluate bone damage, the right ankle joints of rats in different groups were dissected and fixed in 4% paraformaldehyde solution (Servicebio, China) for at least 48 h. Then, all specimens were scanned with micro-computed tomography (Micro-CT). After scanning, all specimens were decalcified by immersion in 14% ethylenediaminetetraacetic acid (EDTA) decalcification solution for 5 days, neutralized in 5% sodium thiosulfate for 3 h, and finally embedded in dehydrated paraffin.

### Micro-computed tomography (Micro-CT) assessments

All specimens were scanned with Micro-CT (Skyscan1172, Bruker, Kontich, Belgium) with a voxel size of 13.1 μm and a rotation step of 0.4, then the dataset was reconstructed using NRecon 1.6.8.0 software (Bruker Co., Kontich, Belgium) to obtain the images of joints. CTvox 2.2.0.0 software (Bruker Co.) was used to reconstruct the three-dimensional (3D) images of the ankle joints (Fig. 3A). To assess the BZXD treatments, the region of interest (ROI) of the bone trabecular of the calcaneus was used as the anatomical site for micro-CT analyses. The ROI was defined by aligning the calcaneus bone along the transaxial plane (Fig. 3A) using Dataviewer, with continuous 50 tomograms of bone trabecular. CTAn 1.11.0.0 (Bruker Co.) software was used to detect and analyze the morphometric parameters of trabecular bone, including Bone Volume / Total Volume, Bone Surface Area / Bone Volume, Trabecular Pattern Factor, Trabecular Number, Trabecular Thickness, Trabecular Separation [40].

### Hematoxylin–Eosin and safranin O-fast green staining

The ankle joint longitudinal Sects. (5–6 μm) were baked in the 60 °C ovens for 1 h, deparaffinized in xylene, hydrated by ethanol, then stained with hematoxylin–eosin (Servicebio, China) and safranin O-fast green (Servicebio, China). Pathological changes were observed based on inflammatory cell infiltration, synovial hyperplasia, angiogenesis, and joint damage by a light microscope.

### LncRNA and mRNA microarray

The total RNA of the synovial tissues was purified by using an RNeasy Mini Kit (Qiagen, Redwood City, CA, USA). RNA quantity and quality were measured using NanoDrop ND-1000, and RNA integrity was assessed by Agilent 2100 Bioanalyzer. Arraystar Rat lncRNA microarrays (v2.0, containing 13611 lncRNAs and 24626 coding transcripts) were used to detect the expression of lncRNAs and mRNAs in a total of 12 rats (3 groups with 4 replicates). According to the Agilent One-Color Microarray-Based Gene Expression Analysis protocol (Agilent Technology), tissue preparation was performed by using Arraystar Flash RNA Labeling Kit to label samples and using Agilent SureHyb for microarray hybridization. After washing, the arrays were scanned by an Agilent DNA Microarray Scanner, analyzed by Agilent Feature Extraction software (version 11.0.0.1) and finally standardized by using Agilent GeneSpring GX v12.1 software. Differentially expressed transcripts (Fold Change  ≥ 2.0 and P < 0.05) were identified by comparing the CIA and Control groups (CIA vs Control) and the CIA + BZXD-treated and CIA groups (BZXD vs CIA). Then, we determined the intersection between the upregulated transcripts in the CIA vs Control groups and the downregulated transcripts in the BZXD vs CIA groups as well as the intersection between the downregulated transcripts in the CIA vs Control groups and the upregulated transcripts in the BZXD vs CIA groups to identify the mechanism by which BZXD reverses pathophysiological changes in CIA.

### LncRNA and mRNA co-expression network

LncRNA exerts its function by positively or negatively acting on target genes at the transcription and post-transcription levels. The function of each lncRNA was based on the function of the connected mRNAs. Therefore, we used Pearson's correlation coefficient (PCC) to calculate the correlation between differentially expressed lncRNAs and mRNAs. According to the set threshold (|PCC|≥ 0.98, P < 0.05), lncRNA‑mRNA pairs were selected to construct the co-expression network and were visualized using Cytoscape 3.7.2 software. Venn diagrams were exerted to exhibit the differentially expressed mRNAs and constructed the key lncRNA and mRNA sub-networks.

### GO and KEGG pathway analysis

To explore the functions of differentially expressed lncRNAs, we performed bioinformatics analysis on the up-regulated and down-regulated lncRNAs after BZXD treatment. Gene Ontology (GO) analysis was performed to determine the biological roles based on the biological processes of the lncRNA‑mRNA pairs, and the Kyoto Encyclopedia of Genes and Genomes (KEGG) pathway was performed to explore the pathways significantly enriched in differentially expressed genes. (DAVID Bioinformatics Resources v6.8, https://david.ncifcrf.gov/) [41]. Use R software ggplot2 package to visualize the enrichment of the KEGG pathway, and draw a chord diagram to visualize the result of GO enrichment through the online website (http://www.bioinformatics.com.cn).

### Quantitative real-time PCR validation

To check the microarray data, we selected 5 differentially expressed lncRNAs and 3 differentially expressed mRNAs across the three groups for verification. The samples remaining after the lncRNA and mRNA microarray were detected for validation by quantitative real-time polymerase chain reaction (RT-qPCR) to verify the reliability of the sequencing data (N = 4). In addition, to verify biological replicates, we examined the expression of differentially expressed lncRNAs and mRNAs in other rat synovial tissues within each group (N = 3). Primers were designed using Primer Premier6 (http://www.premierbiosoft.com/primerdesign/index.html) and were carried out using the Basic Local Alignment Search Tool (BLAST) of NCBI to perform primers to prepare unique amplification products. The method was described in Jingjing Zhang and Jiehua Ma [42]. The primers for each gene are listed in Table 2. The RT-qPCR was performed according to the instructions of the manufacturer by using the SYBR green operational method. The RT-qPCR reaction conditions were as follows: incubation at 95 °C for 10 min, followed by 40 cycles of 95 °C for 10 s and 60 °C for 1 min. The relative expression levels of lncRNAs and mRNAs were calculated in accordance with the cycle threshold (Ct) values and were normalized by the U6 internal parameter using the 2^−ΔΔCt^ method.

**Table 2:** rimers designed for RT-qPCR validation of candidate lncRNAs and mRNAs. Tm: temperature. bp: base pair

Gene name	Forward and reverse primer	Tm (℃)	Product length (bp)
U6	F:5ʹGCTTCGGCAGCACATATACTAAAAT3ʹ R:5ʹCGCTTCACGAATTTGCGTGTCAT3ʹ	60	89
uc.361-	F:5ʹ TGCTCATAAGTGCAAGCTGTC3ʹ R:5ʹ CTGAAAATGTTTGGTATACAAAATC3ʹ	60	128
ENSRNOT00000092834	F:5ʹ TTTATTGACCAATCAGGGAAC3ʹ R:5ʹ TCTGGCTACACTCCTTATTCTG3ʹ	60	86
ENSRNOT00000088010	F:5ʹ TGAGAGAGGCTTGGAAATGG3ʹ R:5ʹ CTCTTGACTGCTGAGCCATCT3ʹ	60	64
ENSRNOT00000089244	F:5ʹ GGAAATGGTAGTGACAAAGGT3ʹ R:5ʹ CCTCCACTATGACAGCATCTAC3ʹ	60	64
ENSRNOT00000084631	F:5ʹGTGTCCACCATCCGACTCATC 3ʹ R:5ʹ CATTTTATCACCCTGCCATCTT3ʹ	60	132
Acvr2a	F:5ʹ TCCTACTCAGGACCCTGGAC 3ʹ R:5ʹ GTGATTAGCCACAGGTCCACA 3ʹ	60	282
Cbx2	F:5ʹ GCTGGTCCTCCAAACACAAC 3ʹ R:5ʹ TCTTTTCCGGTTCTGCACCT 3ʹ	60	113
Morc4	F:5ʹ TTCCTAAAACCTGCGTACAACA 3ʹ R:5ʹ TCCTCCACTTAAGACATTCATCAC3ʹ	60	196

### Fibroblast-like synoviocytes culture and cell transfection

Primary fibroblast-like synoviocytes were isolated from rat synovial tissues. Briefly, the synovial tissue of day 21 CIA rats was taken, and the synovial tissue was washed three times with phosphate-buffered saline. Under sterile conditions, synovial tissue was minced into 2–3 mm pieces and digested with 4 mg/mL type 1 collagenase. Subsequently, the suspension was centrifuged at 500 G for 5 min. The pelleted cells were resuspended in Dulbecco’s modified Eagle’s medium (DMEM) containing 10% fetal bovine serum, 6 mM l-glutamine, 100 units/mL penicillin and 100 µg/mL streptomycin. After overnight incubation, non-adherent cells were removed and adherent cells were cultured in DMEM supplemented with 15% fetal bovine serum. Normal-FLS was isolated from the synovial tissue of day 21 Control rats using the same method. Normal-FLS and CIA-FLS of passages 3–5 were used in the experiments. Plated at a density of 2.0 × 10^5^ cells/mL in a 6-well plate, 2 mL/well, incubated at 37 °C, 5% CO_2_ incubator for 24 h. According to the manufacturer’s instructions, 2.5 μg of shRNA and their NCs were transfected FLSs by using Lipofectamine 3,000 reagent (Invitrogen, Carlsbad, CA, United States). The efficiency of transfections was validated by RT-qPCR. These shRNA sequences are listed in Additional file 1: Table S1.

### Statistical analysis

SPSS (version 26.0; SPSS Inc., Chicago, IL, USA) software was used for statistical analysis. The results were expressed as the mean ± standard deviation (SD). Each time point index difference in the group was analyzed by the analysis of variance of repeated measurement design. Group comparison adopts an independent Student’s t-test or one-way analysis of variance (ANOVA). Spearman’s correlation analysis was used to discover the relationship between lncRNAs and mRNAs. A value of P < 0.05 was considered statistically significant.

## Results

### Major components of BZXD by UPLC–MS/MS analysis

**Fig. 1:**
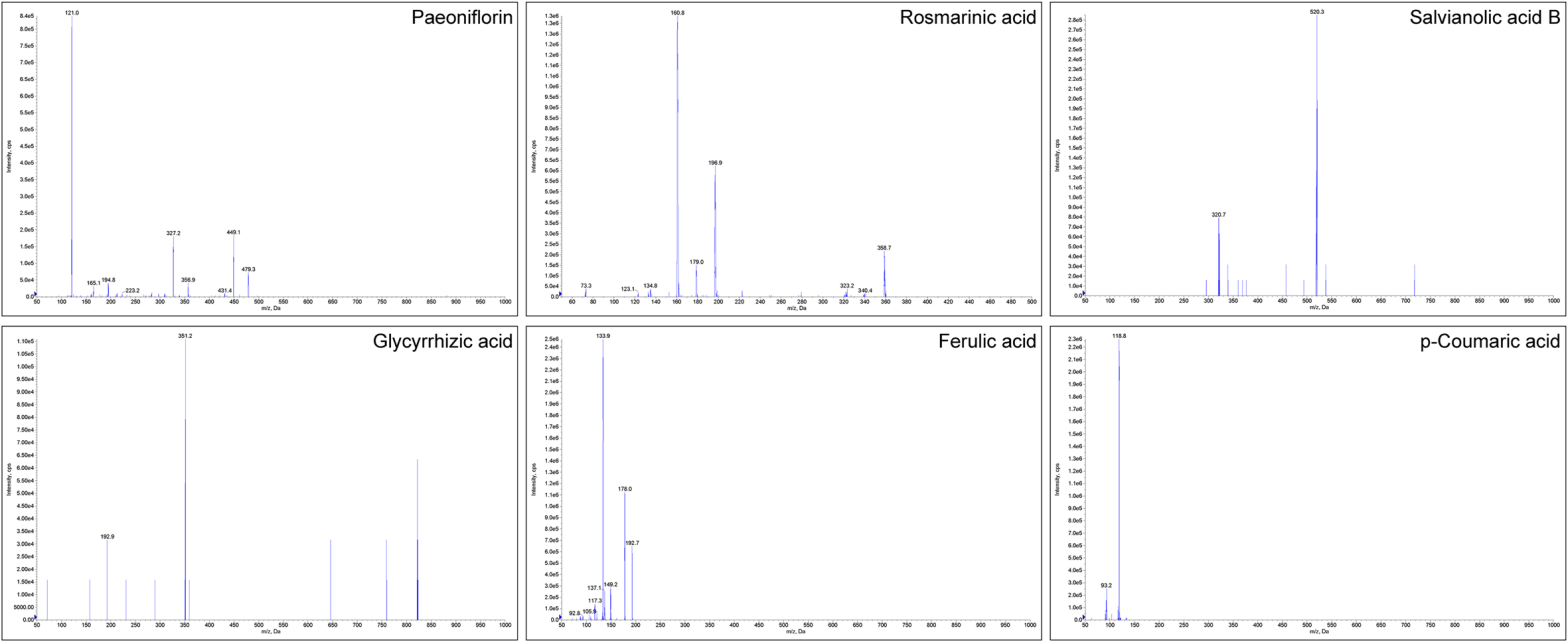
UPLC-MS/MS negative ion secondary mass spectra of BZXD

In the records of “The People’s Republic of China Pharmacopoeia” (2020 edition), the main chemical components of each drug in the BZXD compound were searched, and then the chemical components for the treatment of rheumatoid arthritis were screened out concerning the literature. A total of six chemical components in BZXD were determined, including Paeoniflorin, Rosmarinic acid, Salvianolic acid B, Glycyrrhizic acid, Ferulic acid, and p-Coumaric acid. The quality of BZXD was evaluated by qualitative and quantitative detection of chemical components by using UPLC-MS/MS method. The negative ion mode secondary mass spectrum of the standards was shown in Fig. 1. Three different batches of concentrates were repeated to confirm that all six standards could be detected stably, and the relative content of each standard in BZXD was calculated. The retention times (RT) and contents of the six standards of BZXD were shown in Table 3.

**Table 3:** The retention times and contents of six components in Bi Zhong Xiao decoction

Components	Formula	Fragments (m/z)	RT (min)	Sample RT (min)			Content estimation (ng/mL)		
				S1	S2	S3	S1	S2	S3
Paeoniflorin	C23H28O11	479.400/121.000 Da	2.20	2.21	2.20	2.20	88000.00	85000.00	84500.00
Rosmarinic acid	C18H16O8	359.300/160.900 Da	4.00	4.02	4.01	4.01	14450.00	14500.00	13650.00
Salvianolic acid B	C36H30O16	717.900/520.200 Da	4.34	4.35	4.35	4.35	162500.00	172500.00	158500.00
Glycyrrhizic acid	C42H62O16	821.800/351.300 Da	5.65	5.65	5.65	5.65	11100.00	10550.00	11300.00
Ferulic acid	C10H10O4	193.000/178.100 Da	3.30	3.32	3.32	3.31	555.00	530.00	525.00
p-Coumaric acid	C9H8O3	163.100/118.900 Da	3.04	3.05	3.05	3.04	3230.00	3245.00	3110.00

### BZXD treatment reverse bone erosion in CIA rats

**Fig. 2:**
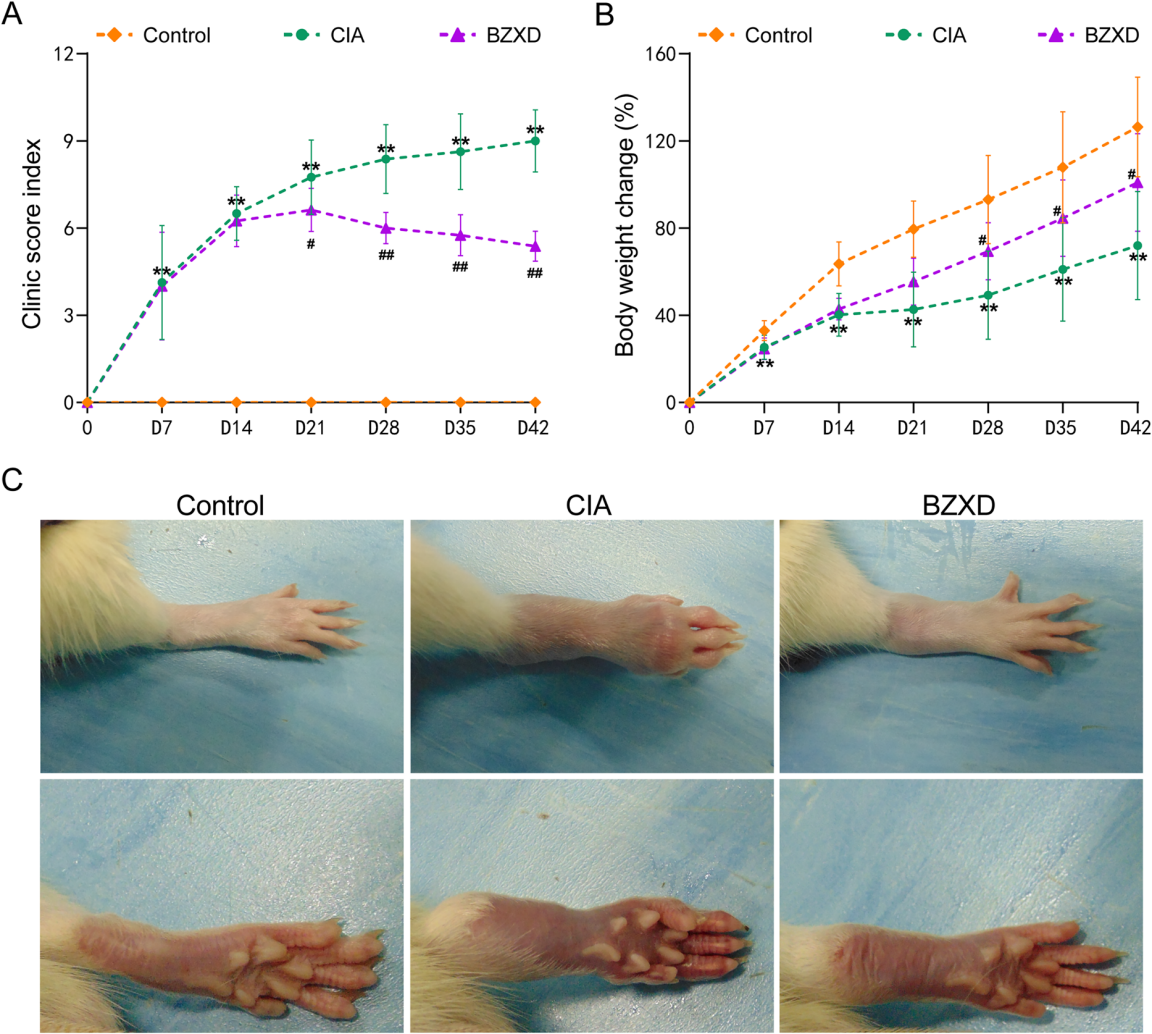
Therapeutic efficacy of BZXD in CIA rats. **A** Clinical scores index. **B** Bodyweight change was measured 0, 7, 14, 21, 28, 35 and 42 days (n = 8 rats per group), two-way repeated-measures ANOVA followed by Fisher’s LSD test, *P < 0.05, **P < 0.01 CIA versus Control; ^#^P < 0.05 ^##^P < 0.01 BZXD versus CIA. **C** Representative image of the right ankle joint of rats

We recorded the clinical score and weight changes of rats and found a significant difference between pre-immunization and postimmunization, which indicated the successful establishment of the CIA animal model. As shown in Fig. 2, the CIA and BZXD groups showed a significant increase in their clinical score, and a significant weight reduction compared with the Control group. From the 14th day, the rats were given BZXD or distilled water by gavage. Compared with the CIA group, the BZXD group showed a significant difference in clinical scores appeared on day 21, while the onset of a significant difference in weight changes was seen on day 28 (Fig. 2A and B). As shown in Fig. 2C, the ankle and toe joints of CIA rats showed more obvious redness, swelling, and hyperemia than in the Control group. However, compared with the CIA group, rats in the BZXD group had less joint swelling, mild heel hyperemia, and slight skin shrinkage.

**Fig. 3:**
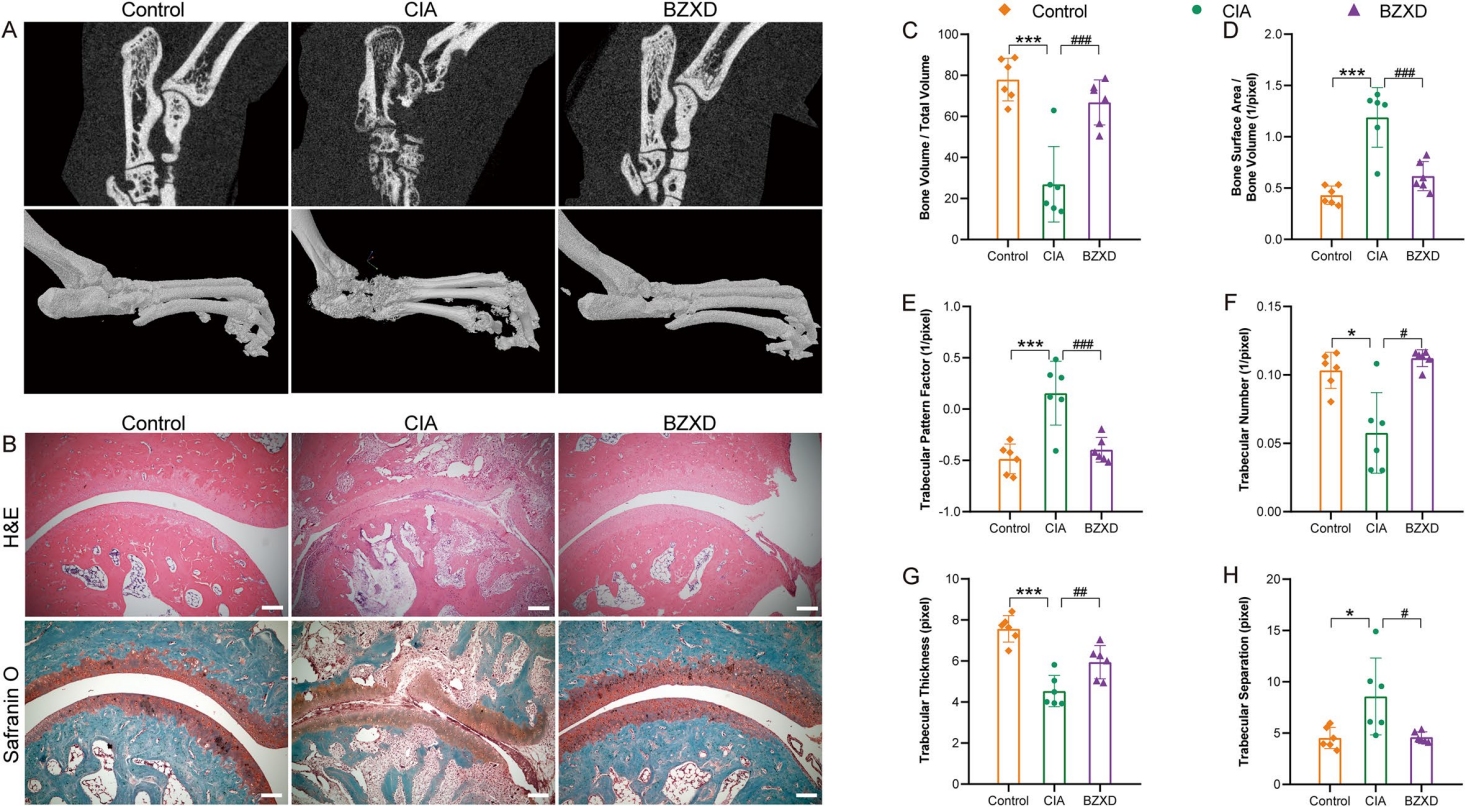
BZXD reverse bone erosion in CIA rats. **A** The bone trabecular of the right calcaneus was chosen as a region of interest (ROI) and representative micro-CT images of the right ankle joints of rats in different groups. **B** Histopathologic changes are in the ankle joint of CIA rats affected by BZXD. Representative images of Hematoxylin–Eosin staining and Safranin O-Fast Green staining (5X) in the ankle joint of rats; scale bar represents 200 μm (n = 6 rats per group). **C**–**H** Quantitative micro-CT analysis of bone trabecular parameters: Bone Volume/Total Volume (**C**), Bone Surface Area/Bone Volume (**D**), Trabecular Pattern Factor (**E**), Trabecular Number (**F**), Trabecular Thickness (**G**), Trabecular Separation (**H**). Data are presented as mean ± SD, n = 6 rats per group, one-way ANOVA followed by Fisher’s LSD test. *P < 0.05, **P < 0.01, ***P < 0.01 CIA versus Control; ^#^P < 0.05, ^##^P < 0.01, ^###^P < 0.001 BZXD versus CIA

To further illustrate that BZXD can reduce inflammation and cartilage destruction, at the end of the study, the ankle joints of rats were sectioned for histological analysis. HE staining in the CIA group showed severe synovial hyperplasia, along with cartilage and bone destruction. The BZXD group displayed a significant effect in reducing synovial hyperplasia and decreased the loss of cartilage compared with the CIA group (Fig. 3B). We also used Safranin O-Fast Green staining to label glycosaminoglycans (GAG) in cartilage tissue to examine cartilage integrity. A significant decrease in Safranin O-zones was observed on the cartilage surface of CIA rats, indicating loss of GAG and cartilage destruction. The cartilage of BZXD rats remained intact and was comparable to that of the Control group. These results demonstrated that BZXD effectively alleviated synovial inflammation and reduced cartilage destructions in CIA rats.

Using micro-CT to scan the ankle joints of Control, CIA, and BZXD rats, we investigated the degree of joint damage in rats. In CIA rats, the articular surfaces of the medial and lateral malleolus are unclear, and the distal tibia, cuboid, navicular, cuneiform, talus, and calcaneus have been severely damaged due to extensive bone erosion. The structure of multiple toe-plantar joints is rough and severely eroded. 28 days after BZXD administration, BZXD rats showed a smooth bone surface, which was closer to the control group, indicating that BZXD effectively reversed bone erosion (Fig. 3A). Quantitative analysis of calcaneal trabecular micro-CT data also showed that BZXD treatment preserved bone quality, which was evident in the morphometric parameters. BZXD treatment was the most efficient in increasing the bone volume / total volume and trabecular thickness while decreasing bone surface area/bone volume and trabecular pattern factor. The values of trabecular number and trabecular separation were similar to those observed in control rats (Fig. 3C–H).

### LncRNA and mRNA microarray analysis

**Fig. 4:**
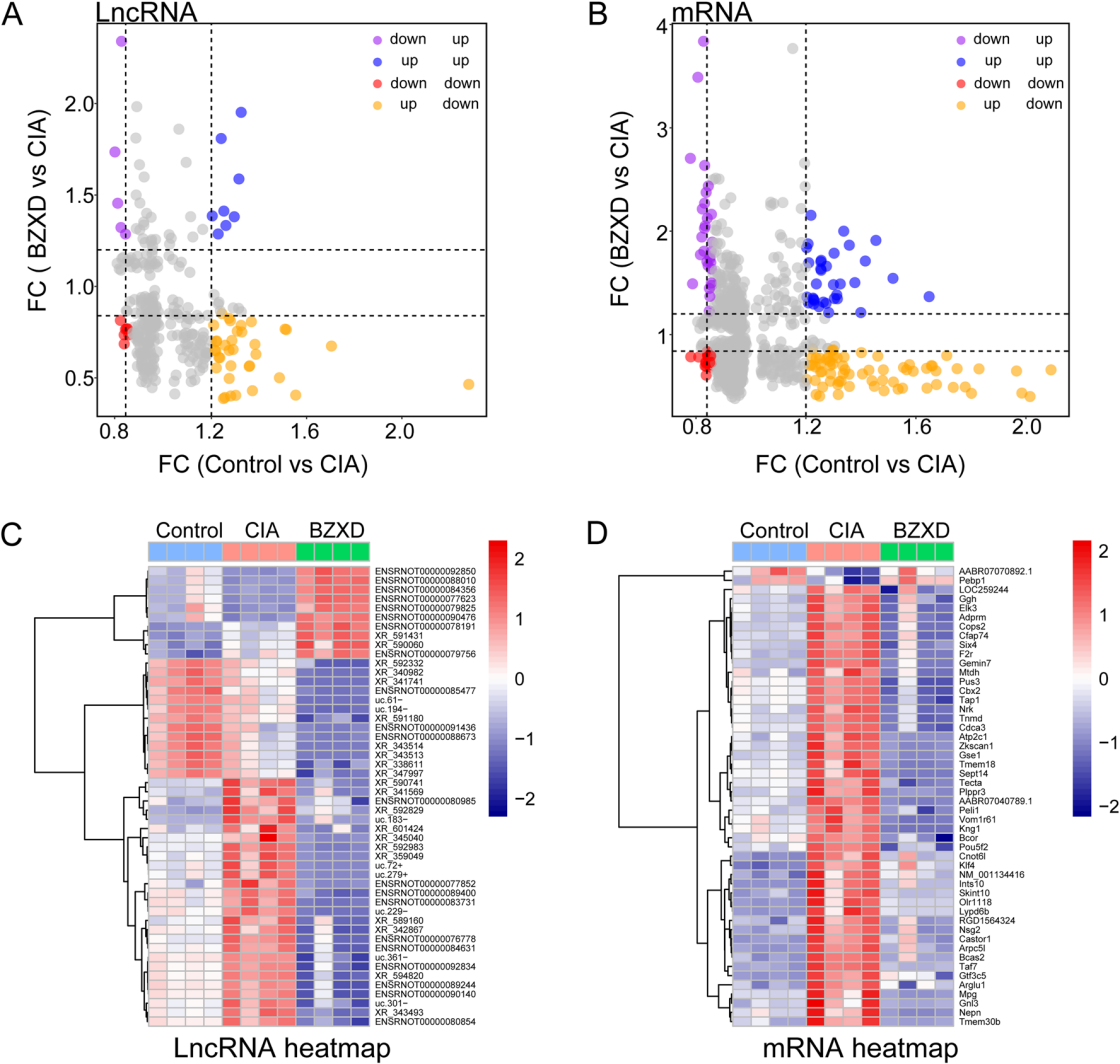
LncRNA and mRNA expression change profiles in different groups. The scatterplots for lncRNAs **A** and mRNAs **B**, respectively. Hierarchical cluster heat map showing top 50 differentially expressed lncRNAs **C** and mRNAs **D** from 2 intersections (CIA vs Control and BZXD vs CIA)

A total of 9,246 lncRNAs and 23,645 mRNAs in the Control, CIA and BZXD groups were detected based on the quality-controlled of the authoritative databases. The scatterplot (Fig. 4A and B) showed the differential expression of lncRNAs and mRNAs in the CIA vs Control and BZXD vs CIA. Differentially expressed lncRNAs and mRNAs were distinguished among the Control, CIA and BZXD groups according to the filtering criteria (P < 0.05, Fold Change  > 2, raw intensity  > 200, RNAlength  < 3 kb). In total, 50 differentially expressed lncRNAs were identified from 2 lncRNA arrays (CIA vs Control and BZXD vs CIA). Among them, 27 common lncRNAs were identified from the intersection of 48 upregulated lncRNAs (CIA vs Control) and 2427 downregulated lncRNAs (BZXD vs CIA) (Table 4). Another 6 common lncRNAs were identified from the intersection of 31 downregulated lncRNAs (CIA vs Control) and 744 upregulated lncRNAs (BZXD vs CIA) (Table 4). 222 differentially expressed mRNAs were found between the Control and CIA groups, and the expression of 9354 mRNAs was significantly altered between the CIA and BZXD groups. Subsequently, 166 differentially expressed mRNAs (including 107 reversely regulated differentially expressed mRNAs) were found among the three groups (Fig. 7A). The top 50 differentially expressed lncRNAs and mRNAs expression patterns are shown in Fig. 4C and D through hierarchical clustering analysis.

**Table 4:** 33 differentially expressed lncRNAs identified from 2 lncRNA arrays (Control vs CIA, BZXD vs CIA)

Transcript_ID	P value		Fold change		Regulation		Chrom	Strand	Relationship
	Control/CIA	BZXD/CIA	Control/CIA	BZXD/CIA	Control/CIA	BZXD/CIA			
ENSRNOT00000077623	0.0454	0.0000	4.07	104.02	Down	up	chr1	−	Exon sense-overlapping
ENSRNOT00000084356	0.0452	0.0000	3.13	70.79	Down	Up	chr18	+	Intergenic
ENSRNOT00000090476	0.0402	0.0000	4.43	39.24	Down	Up	chr3	+	Intergenic
ENSRNOT00000079825	0.0471	0.0000	4.77	32.76	Down	Up	chr2	+	Intergenic
ENSRNOT00000088010	0.0391	0.0000	2.51	25.29	Down	Up	chr3	+	Intergenic
ENSRNOT00000092850	0.0156	0.0000	2.54	18.57	Down	Up	chr12	+	Intron sense-overlapping
uc.361−	0.0000	0.0009	4.89	111.56	Up	Down	chr6	−	Intergenic
ENSRNOT00000092834	0.0001	0.0016	5.51	79.60	Up	Down	chr8	−	Intergenic
ENSRNOT00000089244	0.0016	0.0013	3.81	59.99	Up	Down	chr11	−	Intergenic
ENSRNOT00000090140	0.0014	0.0010	3.86	40.06	Up	Down	chr6	+	Intergenic
ENSRNOT00000076778	0.0001	0.0013	9.05	39.34	Up	Down	chrX	+	Intergenic
ENSRNOT00000084631	0.0009	0.0011	4.49	23.51	Up	Down	chr10	+	Intergenic
XR_594820	0.0000	0.0001	3.57	22.94	Up	Down	chr15	−	Intergenic
XR_343493	0.0023	0.0001	4.11	17.93	Up	Down	chr1	−	Intergenic
uc.183−	0.0000	0.0018	16.59	14.56	Up	Down	chr10	+	Exon sense-overlapping
XR_589160	0.0027	0.0055	4.67	10.53	Up	Down	chr8	+	Intergenic
ENSRNOT00000080854	0.0031	0.0002	2.40	9.53	Up	Down	chr8	−	Intergenic
ENSRNOT00000083731	0.0066	0.0000	2.26	6.85	Up	Down	chr8	−	Intergenic
uc.229−	0.0005	0.0000	2.26	6.74	Up	Down	chr4	−	Intergenic
XR_590741	0.0002	0.0001	3.93	6.16	Up	Down	chr1	+	Intergenic
ENSRNOT00000089400	0.0016	0.0000	2.12	5.92	Up	Down	chr20	−	Intergenic
uc.301−	0.0017	0.0009	2.45	5.87	Up	Down	chr1	−	Intronic antisense
XR_342867	0.0116	0.0043	2.24	5.83	Up	Down	chr1	+	Intergenic
XR_592829	0.0004	0.0045	8.76	5.54	Up	Down	chr6	+	Intergenic
uc.279 +	0.0042	0.0001	2.53	4.92	Up	Down	chr3	−	Intron sense-overlapping
XR_359049	0.0018	0.0001	2.42	4.32	Up	Down	chr13	+	Intergenic
XR_592983	0.0000	0.0000	2.41	4.23	Up	Down	chr6	−	Intronic antisense
uc.72 +	0.0044	0.0002	2.17	3.70	Up	Down	chr3	+	Intronic antisense
XR_601424	0.0150	0.0075	2.60	3.69	Up	Down	chr6	+	Exon sense-overlapping
ENSRNOT00000080985	0.0034	0.0057	2.84	3.38	Up	Down	chr17	+	Intergenic
XR_345040	0.0237	0.0023	2.03	3.27	Up	Down	chr3	+	Intergenic
ENSRNOT00000077852	0.0040	0.0001	2.05	2.94	Up	Down	chr1	−	Intergenic
XR_341569	0.0022	0.0069	2.31	2.61	Up	Down	chr17	+	Intergenic

### LncRNA-mRNA co-expression network

**Fig. 5:**
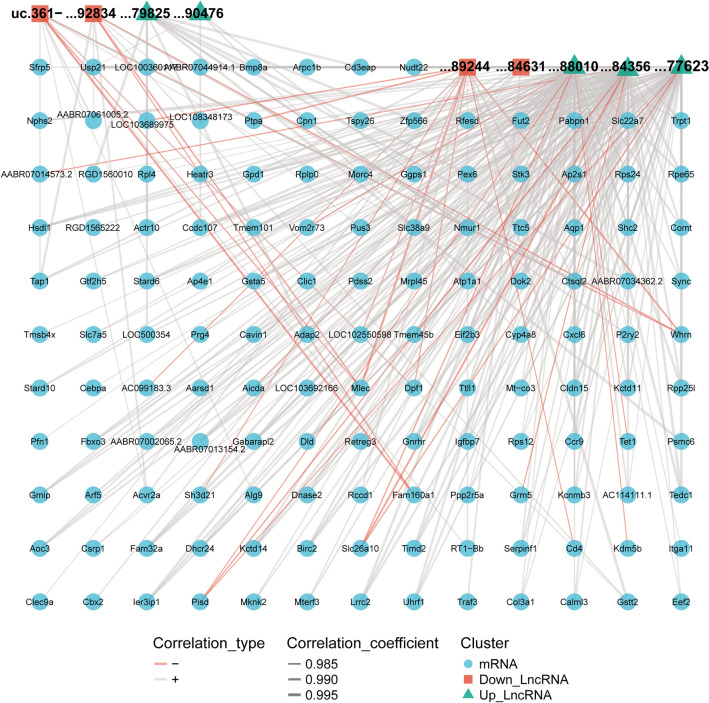
Co-expression network of differentially expressed lncRNAs and mRNAs following BZXD treatment. Upregulated in BZXD vs CIA and downregulated in CIA vs Control lncRNAs are labeled in green, downregulated in BZXD vs CIA and upregulated in CIA vs Control lncRNAs are labeled in red, and mRNAs are labeled in blue

LncRNAs are involved in a variety of diseases and biological processes, but the functions of lncRNAs remain largely unknown. The co-expression network of lncRNA and mRNA plays an important role in the preliminary prediction of lncRNA function and provides evidence for the regulatory mechanism of lncRNA in biological functions [23]. According to the Fold Change value of BZXD vs CIA, from the 33 reverse differentially expressed lncRNAs, 9 lncRNAs were selected as potential key lncRNAs. Then, we constructed a co-expression network based on the selected lncRNAs normalized expression data and all the difference expressed mRNAs normalized data. The co-expression network was composed of 9 lncRNA nodes, 138 mRNA nodes and 302 edges (Fig. 5).

### KEGG pathway and GO analysis of lncRNAs

**Fig. 6:**
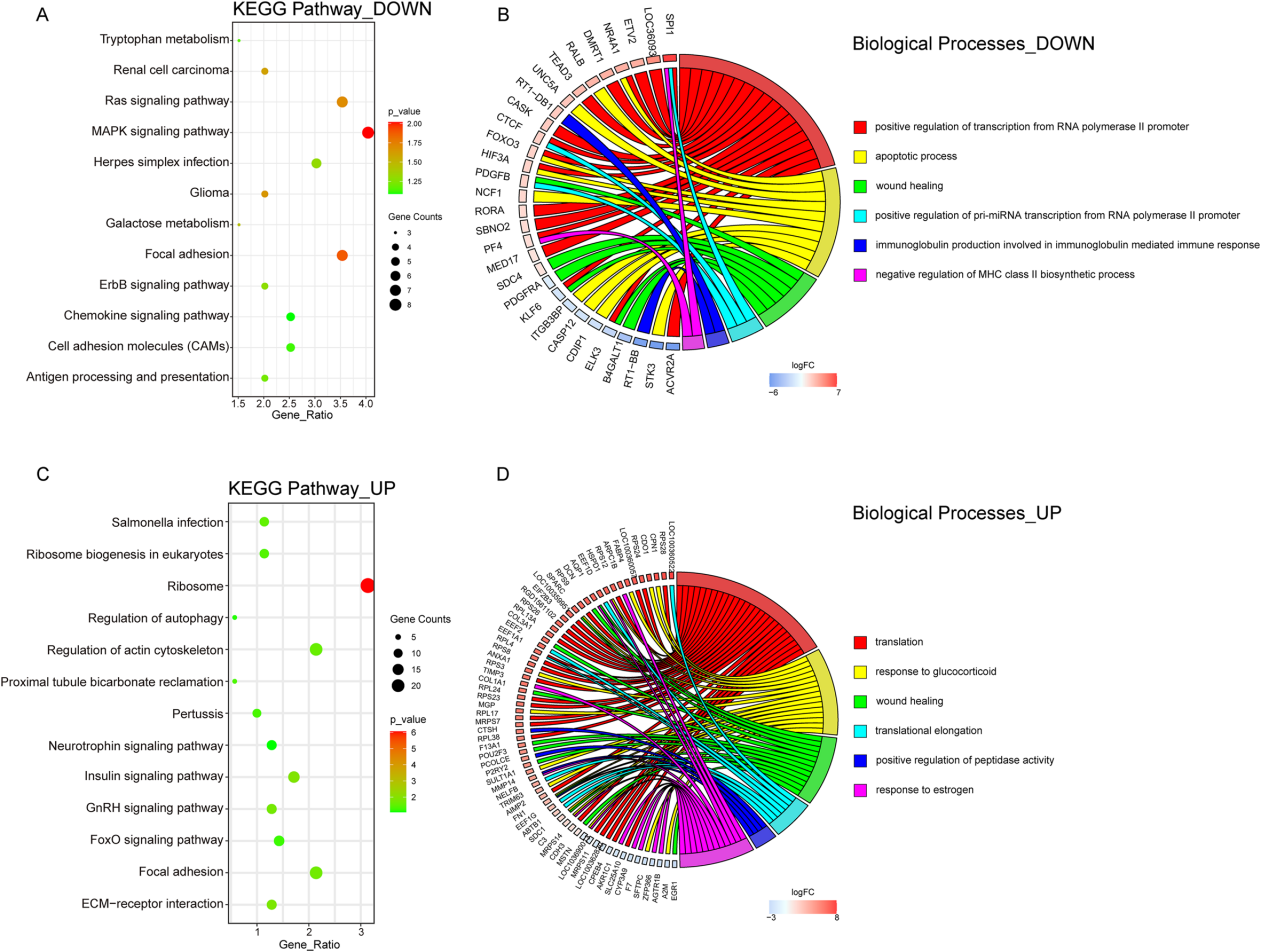
KEGG Pathway and Biological Processes enrichment analysis of differentially expressed lncRNAs. **A** and **C** The enriched KEGG pathway items of the dysregulated lncRNAs. **B** and **D** Top 6 significant GO terms in Biological Processes. UP represents the upregulated in BZXD vs CIA and downregulated in CIA vs Control lncRNAs **A** and **B**, DOWN represents downregulated in BZXD vs CIA and upregulated in CIA vs Control lncRNAs **C** and **D**

The lncRNA-mRNA co-expression correlation analysis facilitates functional annotation of uncharacterized lncRNA. Therefore, we constructed the co-expression network to group lncRNAs and mRNAs into functionally related sets that allow predictions of the roles of lncRNAs. Then, we used DAVID to perform the KEGG pathway and GO analysis to reveal the functions of BZXD up-regulating lncRNAs and BZXD down-regulating lncRNAs, respectively. GO enrichment analysis of BZXD down-regulating lncRNAs revealed that were related to the apoptotic process, positive regulation of transcription from RNA polymerase II promoter, immunoglobulin production involved in immunoglobulin mediated immune response and negative regulation of MHC class II biosynthetic process (Fig. 6B). The top enriched biological process of BZXD up-regulating lncRNAs included the response to glucocorticoid, translation and positive regulation of peptidase activity (Fig. 6D). The most enriched pathways of BZXD down-regulating lncRNAs were MAPK signaling pathway, Focal adhesion and Ras signaling pathway. ECM-receptor interaction, Regulation of actin cytoskeleton and FoxO signaling pathway were mainly enriched in KEGG pathways of BZXD up-regulating lncRNAs. All significant KEGG pathways were listed in Fig. 6A and C suggesting BZXD may reverse bone erosion in CIA rats by regulating these pathways.

### Key lncRNA‑mRNA co-expression sub‑network

**Fig. 7:**
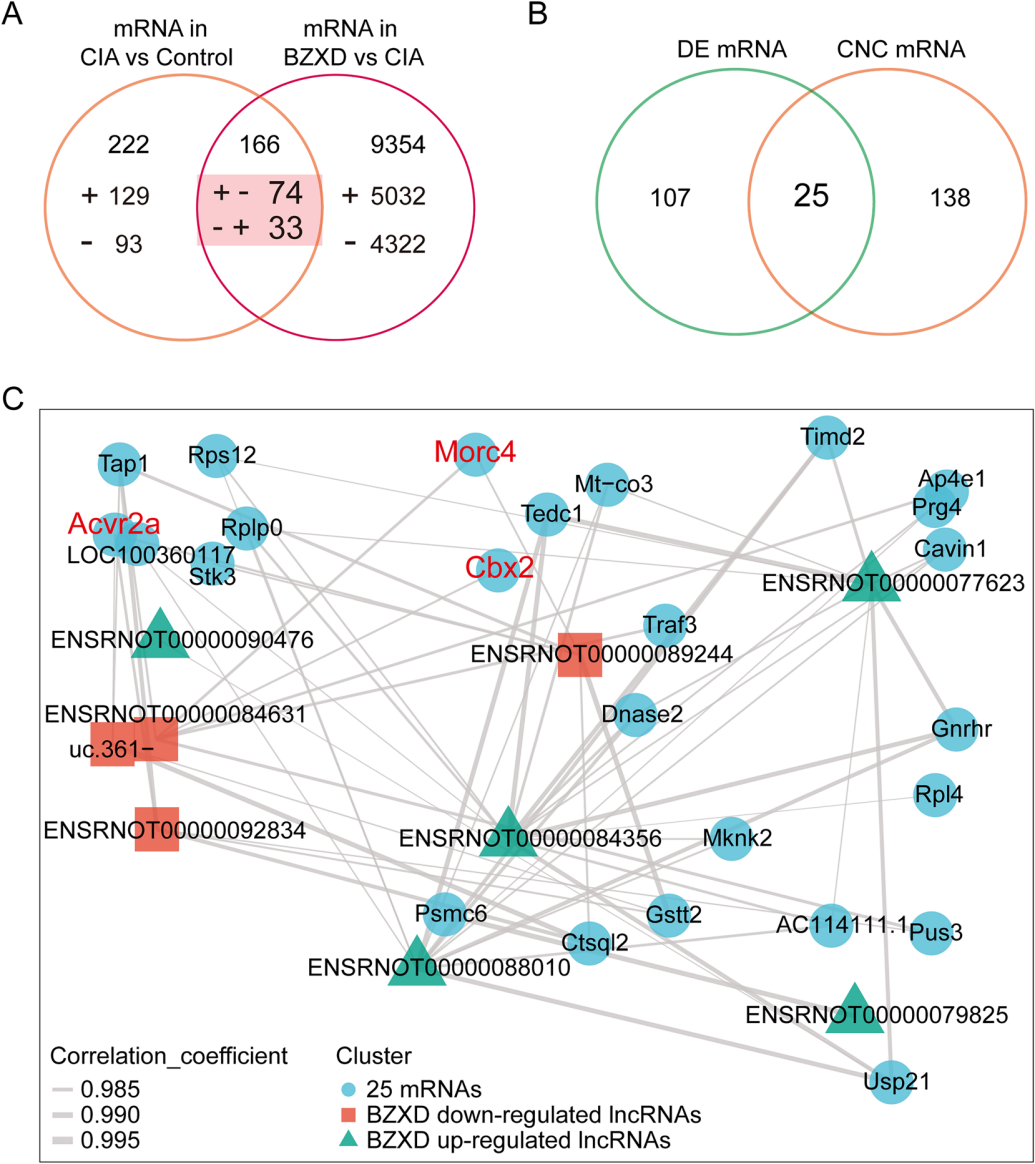
Key lncRNA‑mRNA co-expression sub‑network. **A** Venn diagram of mRNAs from CIA vs Control and BZXD vs CIA. The “ + ” indicates upregulated mRNAs, while the “−” indicates downregulated mRNAs. The “ + −” indicates mRNAs are upregulated in CIA vs Control groups but downregulated in BZXD vs CIA groups. The “− + ” indicates mRNAs are downregulated in CIA vs Control groups but upregulated in BZXD vs CIA groups. **B** Venn diagram of reverse differentially expressed mRNAs in 2 intersections (CIA vs Control and BZXD vs CIA) and mRNAs from co-expression network. **C** Co-expression sub‑network consists of 9 lncRNAs and 25 mRNAs

Through the co-expression network in Fig. 5, we found that one lncRNA can target up to 90 coding genes. Although co-expression analysis is a powerful method for exploring the function of lncRNA, it always generates large amounts of lncRNA-mRNA pairs including many useless pairs. Therefore, we tried to find the core mRNA through the Venn diagram to reduce the co-expression pairs of lncRNA-mRNA. As shown in Fig. 7A, we obtained 107 reverse differentially expressed LncRNAs from the intersection of the two sets (CIA vs Control and BZXD vs CIA). The 25 core mRNAs were obtained by the intersection of 107 reverse differentially expressed mRNAs and 138 mRNAs participating in the co-expression network (Fig. 7B). The relationship pairs of these 25 core mRNAs were extracted from the lncRNA-mRNA co-expression network to obtain the key lncRNA-mRNA co-expression sub-network which consists of 4 lncRNAs up-regulated by BZXD, 5 lncRNAs down-regulated by BZXD, and 25 core mRNAs (Fig. 7C).

### Validation of lncRNAs and mRNAs

**Fig. 8:**
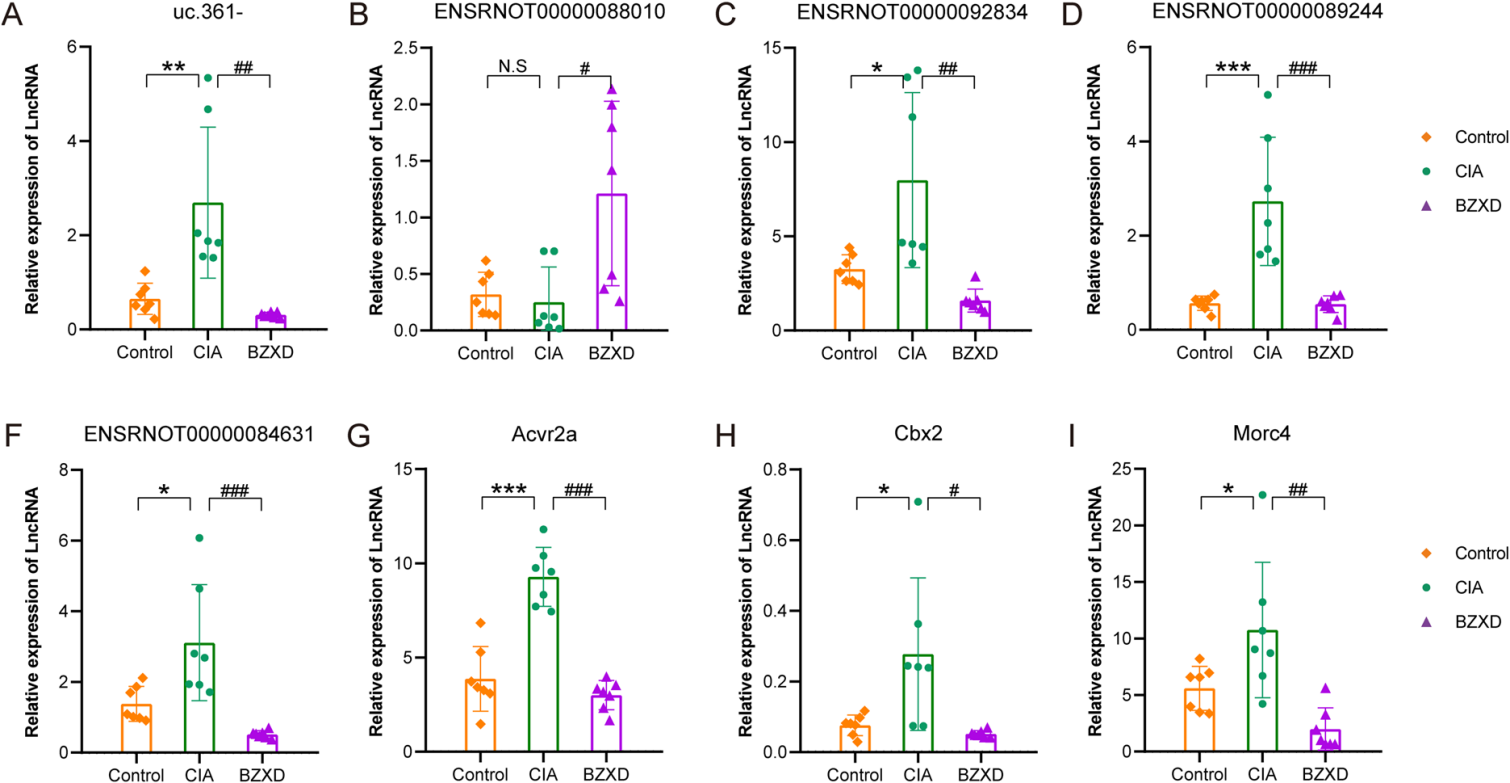
Validation of differentially expressed lncRNAs and mRNAs by RT-qPCR. Data are presented as mean ± SD, n = 7 rats per group, student's t-test. *P < 0.05, **P < 0.01, ***P < 0.001 CIA versus Control; ^#^P < 0.05, ^##^P < 0.01, ^###^P < 0.001 BZXD versus CIA

According to the fold change and functional annotation, five differentially expressed lncRNAs (uc.361-, ENSRNOT00000092834, ENSRNOT00000088010, ENSRNOT00000089244, ENSRNOT00000084631) and three differentially expressed mRNAs (Acvr2a, Cbx2, Morc4) were selected to validate the expression changes in the microarray data by RT-qPCR. Both RNA-seq and RT-qPCR showed the expression of uc.361−, ENSRNOT00000092834, ENSRNOT00000089244, ENSRNOT00000084631, Acvr2a, Cbx2, and Morc4 were upregulated in the CIA group but downregulated after BZXD treatment and that ENSRNOT00000088010 expression decreased after CIA but increased after BZXD treatment. Although the RT-qPCR results of lncRNA ENSRNOT00000088010 showed that the comparison between the control group and the CIA group was not statistically significant, its expression trend was consistent with RNA-seq (Fig. 8). In total, RT-qPCR validation indicated good reliability and reproducibility of the lncRNAs and mRNAs changes determined by RNA-seq.

### In vitro functional validation

**Fig. 9:**
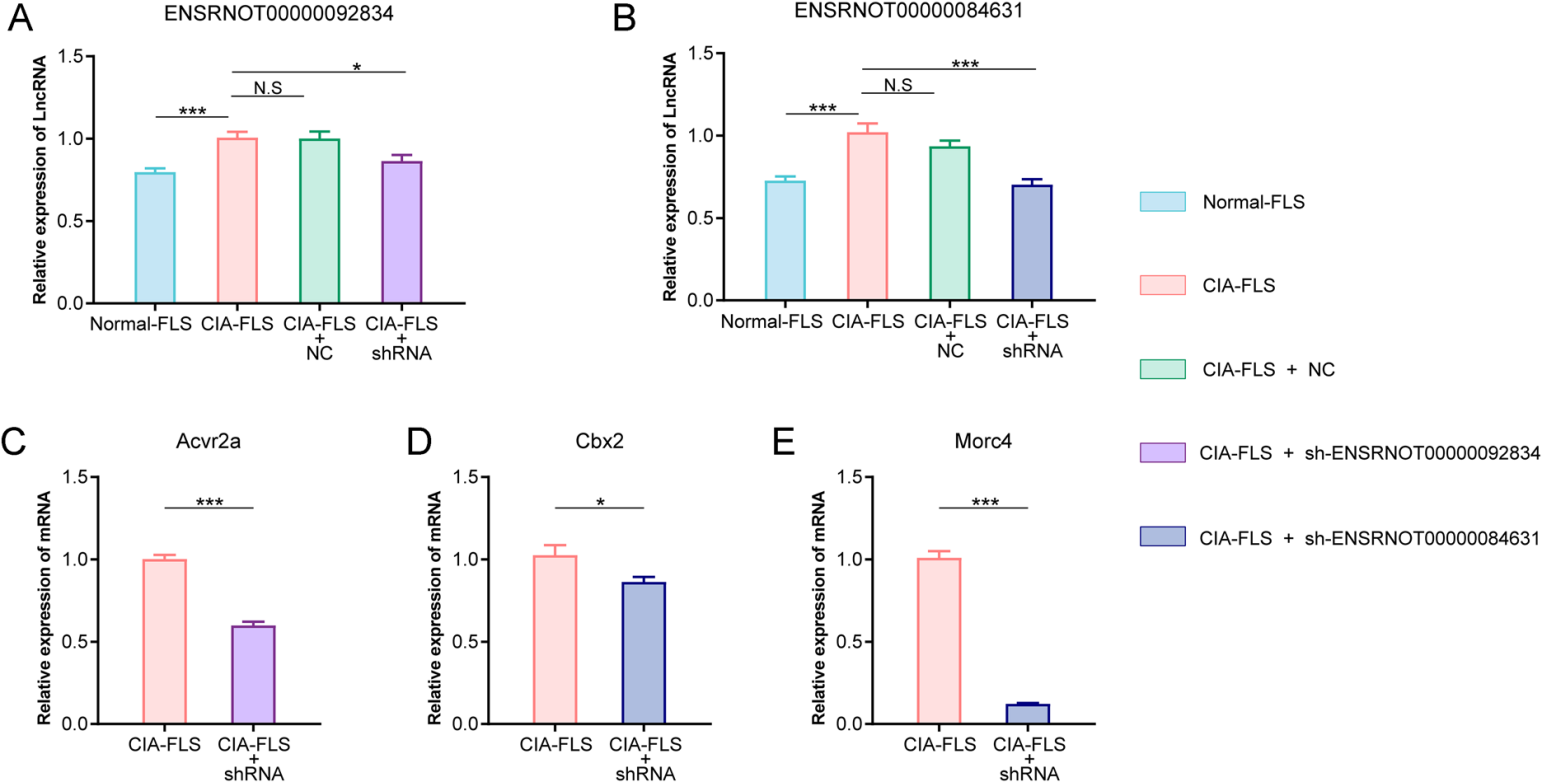
In vitro functional validation of differentially expressed lncRNAs by RT-qPCR. Data are presented as mean ± SD, n = 4 rats per group, student's t-test. *P < 0.05, **P < 0.01, ***P < 0.001

In addition, to verify the relationship between lncRNAs and their target genes, we selected the lncRNAs with the highest correlation coefficient with mRNAs for verification (ENSRNOT00000092834-Acvr2a, pcc = 0.989; ENSRNOT00000084631-Cbx2, pcc = 0.985; ENSRNOT00000084631-Morc4, pcc = 0.988). As shown in Fig. 9A and B, the expression of lncRNAs ENSRNOT00000092834 and ENSRNOT00000084631 in FLSs was consistent with that in synovial tissue: compared with the Control group, they were all upregulated in the CIA group. Furthermore, sh-ENSRNOT00000092834 and sh-ENSRNOT00000084631 remarkably lowered the expression of ENSRNOT00000092834 and ENSRNOT00000084631 in CIA-FLSs, which were adopted for further experiments. Silencing ENSRNOT00000092834 decreased the expression of Acvr2a in CIA-FLS (Fig. 9C) and silencing ENSRNOT00000084631 significantly decreased Cbx2 and Morc4 levels (Fig. 9D and E).

## Discussion

During RA progression, the synovium transforms into a proliferative, aggressive tissue that destroys cartilage and bone [43], and if not treated in time, it will eventually lead to disability. However, most studies of RA treatment strategies have focused on reducing inflammatory cell infiltration and synovial hyperplasia, and less attention has been paid to inhibiting the pathogenesis of cartilage damage. In our previous animal experiments, we found that BZXD treatment could significantly inhibit bone erosion in RA. Therefore, this study intends to explore the pathogenic mechanism of bone erosion in CIA rats and the protective effect of BZXD on joints from the perspective of lncRNA and mRNA. In this study, we used HE and safranin fast green staining, and Micro-CT to prove that BZXD can indeed effectively reverse bone erosion in CIA rats. The lncRNA and mRNA expression profiles were obtained by high-throughput gene chip detection of the Control, CIA and BZXD groups. We screened out the differentially expressed lncRNAs and mRNAs, which were both differentially expressed genes in the CIA vs Control group and BZXD vs CIA group, and the expression trends in the CIA vs Control group and BZXD vs CIA group were opposite (indicating that BZXD can regulate its expression). A co-expression network was constructed for differentially expressed lncRNAs and mRNAs, and in-depth bioinformatics analysis was performed.

To understand the biological processes and signaling pathways involved in these differentially expressed lncRNAs, GO functional and KEGG pathway enrichment analyses were performed. GO analysis revealed that the differentially expressed lncRNAs up-regulated in CIA rats were mainly enriched in the immune response, such as immunoglobulin production involved in immunoglobulin mediated immune response and negative regulation of MHC class II biosynthetic process. The main causes of RA synovial inflammation are immune activation and signal transduction, leading to arthritis and arthralgia [44]. Our study shows that BZXD can effectively inhibit the development of RA by regulating these processes. KEGG pathway analysis showed that ECM-receptor interaction, Focal adhesion and FoxO signaling pathways were the main enrichment pathways for the differentially expressed lncRNAs down-regulated in CIA rats. Articular cartilage damage is a very important pathological change in the process of RA joint destruction. Cartilage tissue is composed of extracellular matrix (ECM) synthesized by chondrocytes. Degradation of the cartilaginous ECM provides an opportunity for the hyperplastic synovium to directly invade the subchondral bone [45]. The self-repairing ability of cartilage tissue is limited, and BZXD can promote its repair process by inhibiting the ECM receptor gene. In addition, differentially expressed lncRNAs up-regulated in CIA rats were involved in MAPK signaling, Focal adhesion, Ras signaling, Antigen processing and presentation, and Chemokine signaling, which are closely related to RA disease. Under normal physiological conditions, MAPK signaling pathways were involved in the regulation of differentiation of healthy cells and normal cell proliferation, and survival [46]. However, the TLR-dependent MAPK pathway activation mediates the release of proinflammatory cytokines from RA synovial fibroblasts and macrophages, thereby promoting persistent inflammation and joint damage [47]. Studies have shown that RA synovial tissue can inhibit the proliferation of osteoblasts through the MAPK pathway [48], so we speculate that BZXD can reverse bone erosion in CIA rats by regulating the MAPK pathway. A transcriptomic study of the tarsal joints demonstrated that “MAPK signaling”, “focal adhesion” and “chemokine signaling” contribute to the migration and maturation of osteoclasts and osteoblasts. These pathways were also significantly enriched in our current study, suggesting that they were involved in the process of bone remodeling starting in early synovial tissue and progressing gradually [45]. Overall, we found that BZXD could regulate these signaling pathways, which helped us understand the mechanism of BZXD reverse bone erosion in CIA rats.

Joint destruction is a direct cause of disability in RA patients, and previous studies have shown that joint destruction is directly related to chondrocytes, osteoblasts, and osteoclasts. In addition, the production of osteoclasts from synovial cell cultures from RA patients showed that fibroblasts and osteoclast precursor cells supporting osteoclastogenesis were present in the RA synovial tissue [49]. Consistently, we found 3 mRNAs involved in joint destruction from transcriptomic analysis of rat synovial tissue in the present study. In a recent review in Nature Reviews Rheumatology, Noriko Komatsu [50] also concluded that FLSs play an important role in joint injury by enhancing osteoclastic bone erosion and cartilage destruction and inhibiting osteoblastic bone formation. Therefore, we chose FLSs for further study.

Acvr2a, a member of the activin A type II receptor, binds activin A with high affinity but bone morphogenetic protein (BMP) with relatively low affinity [51]. Activin A and BMP both are a member of the transforming growth factor-beta growth factor superfamily. Studies have shown that activin A can inhibit BMP signaling by binding to Acvr2a and Acvr2b [52]. Notably, activin A mediates activin receptor signaling primarily through Acvr2a [53]. Activin A stimulates osteoclastogenesis and inhibits osteoblast formation to regulate multiple biological functions, such as inflammation and bone homeostasis [52]. Imbalances in bone homeostasis can lead to bone loss, which ultimately manifests in significant changes in bone structure [54]. In our study, compared with the BZXD group, bone erosion was more obvious in the CIA group. The results of the microarray chip and RT-qPCR showed that Acvr2a was up-regulated in the synovial tissue of CIA rats, and BZXD down-regulated the expression of Acvr2a. Additionally, we observed that the BZXD-regulated differentially expressed lncRNA ENSRNOT00000092834, which was silenced in CIA-FLSs, downregulated the expression of Acvr2a. Consistently, one study reported that miR-21-5p inhibited osteoclast differentiation by downregulating Acvr2a, effectively attenuating bone loss in OVX-induced osteoporotic mice [55]. Based on these findings, we speculate that BZXD may inhibit bone erosion by regulating the expression of uc.361−, ENSRNOT00000092834and Acvr2a impact on osteoblast and osteoclast differentiation. However, the specific regulatory mechanism needs to be further studied.

Microrchidia (MORC family CW zinc finger proteins) is a highly conserved nuclear protein superfamily consisting of Morc1, Morc2, Morc3 and Morc4. The Morc4 protein contains a CW zinc finger motif, a HATPase-c domain, a coiled-coil region, a nuclear localization signal, and a nuclear matrix binding domain, and is considered a transcriptional regulator that plays an important role in regulating biological processes [56]. Morc4 is widely expressed at low levels in healthy tissues but is highly expressed in diseases such as acute and chronic pancreatitis, breast cancer cells, and diffuse large B-cell lymphoma [57–60]. Studies have reported that overexpression of Morc4 increases breast cancer cell viability, migration and invasion, and reduces apoptosis in various breast cancer cell lines [60, 61]. Morc4 has not been studied in RA, but our study shows that Morc4 was also overexpressed in the synovial tissue of CIA rats. The homologous protein MORC3, as a transcriptional regulator, plays a key role in maintaining bone remodeling by participating in STAT, Akt and MAPK signaling pathways [62, 63]. One study revealed that Morc3^mut±^ mice increased trabecular bone mass by inhibiting osteoclastogenesis and bone resorption and promoting osteoblast differentiation [64]. In this study, BZXD decreased the expression of Morc4, and Micro-CT showed that BZXD increased trabecular thickness and reduced trabecular pattern factor. Therefore, we speculate that Morc4 may also play an important role in maintaining bone remodeling during the pathological process of RA. In addition, it was observed in our in vitro experiments that Morc4 was significantly downregulated after silencing the expression of the BZXD-regulated differentially expressed lncRNA ENSRNOT00000084631 in CIA-FLSs. BZXD may regulate ENSRNOT00000084631 and Morc4 expression in synovial fibroblasts to inhibit osteoclastogenesis and inhibit RA bone destruction at the initial stage.

ChromoBox (CBX) protein includes eight members whose epigenetic gene repression is important for the maintenance and renewal capacity of pluripotent stem cells [65]. Cbx2, Cbx4, Cbx6, Cbx7, and Cbx8 make up canonical polycomb repressive complex 1 (PRC1) complexes. As a polycomb protein, Cbx2 regulates gene transcription by maintaining a chromatin state, thereby directing various biological processes [66]. Gene transcriptome meta-analysis reported higher expression of Cbx2 in many tumors including colon, breast, stomach and lung compared to normal tissues [67]. In our study, the Cbx2 mRNA level was highly expressed in the synovial tissue of CIA rats compared with normal rats. The key lncRNA‑mRNA sub‑network analysis indicated that Cbx2 was positively correlated with lncRNA ENSRNOT00000084631, we also determined by RT-qPCR that the expression of Cbx2 decreased after silencing ENSRNOT00000084631. Studies have shown that Cbx2 knockdown reduced cancer cell proliferation, migration, and invasion [68–70]. In addition, studies also have found that Cbx2 can inhibit osteoblast proliferation while knocking down its expression increases the expression of bone-related markers [71, 72]. Our study suggested that BZXD decreased the mRNA level of Cbx2. Compared with the CIA group, BZXD significantly increased the relative volume of trabecular bone, the thickness of trabecular bone, and the number of trabecular bones as the main markers of bone mass. The key lncRNA‑mRNA sub‑network analysis indicated that Cbx2 was positively correlated with lncRNA ENSRNOT00000084631. These findings indicated that BZXD could be involved in the regulation of ENSRNOT00000084631 and Cbx2 mRNA expression. In summary, BZXD may block the effect of Cbx2 on inhibiting osteoblast differentiation by regulating lncRNA ENSRNOT00000084631, which may be one of the mechanisms by which BZXD improves bone erosion in CIA rats.

## Conclusions

In summary, this study elucidates the possible mechanism of BZXD reversing bone erosion in CIA rats from the perspective of lncRNA and mRNA. Uncovering possible mechanisms of joint destruction in rheumatoid joints from synovial tissue and tissue-destructive synovial fibroblast-associated bone homeostasis. At the same time, we found 2 lncRNAs and 3 mRNAs that are expected to be potential therapeutic targets for RA. Meanwhile, the roles and molecular mechanisms of these specific lncRNAs in regulating mRNA should be further investigated.

## Supplementary Information

**Additional file 1: Table S1.** shRNA sequences (shRNA: short hairpin RNA).

## Data Availability

The data included in this investigation are available from the corresponding author.
