# Supplementary material for: An intersectional analysis of LncRNAs and mRNAs reveals the potential therapeutic targets of Bi Zhong Xiao Decoction in collagen-induced arthritis rats
Source: Chin Med. 2022 Sep 16;17:110. doi: 10.1186/s13020-022-00670-z (PMC9479270; doi:10.1186/s13020-022-00670-z)
Supplement: Supplementary file 1 — Additional file 1: Table S1. shRNA sequences (shRNA: short hairpin RNA). [file 13020_2022_670_MOESM1_ESM.docx]

| **Oligo Sequence** | | |
| --- | --- | --- |
| sh-ENSRNOT00000092834 #1 | Sense | 5'-CACCGCAGAGATCAGCATTTGAATAATTCAAGAGATTATTCAAATGCTGATCTCTGTTTTTTG-3' |
|  | Antisense | 5'-GATCCAAAAAACAGAGATCAGCATTTGAATAATCTCTTGAATTATTCAAATGCTGATCTCTGC-3' |
| sh-ENSRNOT00000092834 #2 | Sense | 5'-CACCGTGGTGGGAATCAAACCTGAGTTTCAAGAGAACTCAGGTTTGATTCCCACCATTTTTTG-3' |
|  | Antisense | 5'-GATCCAAAAAATGGTGGGAATCAAACCTGAGTTCTCTTGAAACTCAGGTTTGATTCCCACCAC-3' |
| sh-ENSRNOT00000092834 #3 | Sense | 5'-CACCGTGCACCATGTATATGCTGGTTTCAAGAGAACCAGCATATACATGGTGCACTTTTTTG-3' |
|  | Antisense | 5'-GATCCAAAAAAGTGCACCATGTATATGCTGGTTCTCTTGAAACCAGCATATACATGGTGCAC-3' |
| sh-ENSRNOT00000084631 #1 | Sense | 5'-CACCGCTCGGGAAAGTGTTTATAAATTCAAGAGATTTATAAACACTTTCCCGAGCTTTTTTG-3' |
|  | Antisense | 5'-GATCCAAAAAAGCTCGGGAAAGTGTTTATAAATCTCTTGAATTTATAAACACTTTCCCGAGC-3' |
| sh-ENSRNOT00000084631 #2 | Sense | 5'-CACCGGGAGAGGAAAGAAGCGAAACTTCAAGAGAGTTTCGCTTCTTTCCTCTCCCTTTTTTG-3' |
|  | Antisense | 5'-GATCCAAAAAAGGGAGAGGAAAGAAGCGAAACTCTCTTGAAGTTTCGCTTCTTTCCTCTCCC-3' |
| sh-ENSRNOT00000084631 #3 | Sense | 5'-CACCGGATTCGCCTGGTGGGAATAATTCAAGAGATTATTCCCACCAGGCGAATCCTTTTTTG-3' |
|  | Antisense | 5'-GATCCAAAAAAGGATTCGCCTGGTGGGAATAATCTCTTGAATTATTCCCACCAGGCGAATCC-3' |

**Supplementary Table S1: shRNA sequences** (shRNA: short hairpin RNA).
